# Supplementary material for: Variant-to-gene mapping followed by cross-species genetic screening identifies GPI-anchor biosynthesis as a regulator of sleep
Source: Sci Adv. 2023 Jan 6;9(1):eabq0844. doi: 10.1126/sciadv.abq0844 (PMC9821868; doi:10.1126/sciadv.abq0844)
Supplement: Supplementary file 1 — Figs. S1 to S5 [file sciadv.abq0844_sm.pdf]

Science Advances
AAAS

Supplementary Materials for

Variant-to-gene mapping followed by cross-species genetic screening identifies GPI-anchor biosynthesis as novel regulator of sleep

Justin Palermo *et al.*

Corresponding author: Phillip R. Gehrman, gehrman@upenn.edu; Struan F.A. Grant, grants@chop.edu; Alex C. Keene, keenea@tamu.edu

Sci. Adv. **9**, eabq0844 (2023)
DOI: 10.1126/sciadv.abq0844

The PDF file includes:

Figs. S1 to S5
Legends for tables S1 to S4

Other Supplementary Material for this manuscript includes the following:

Tables S1 to S4

Supplementary Figures

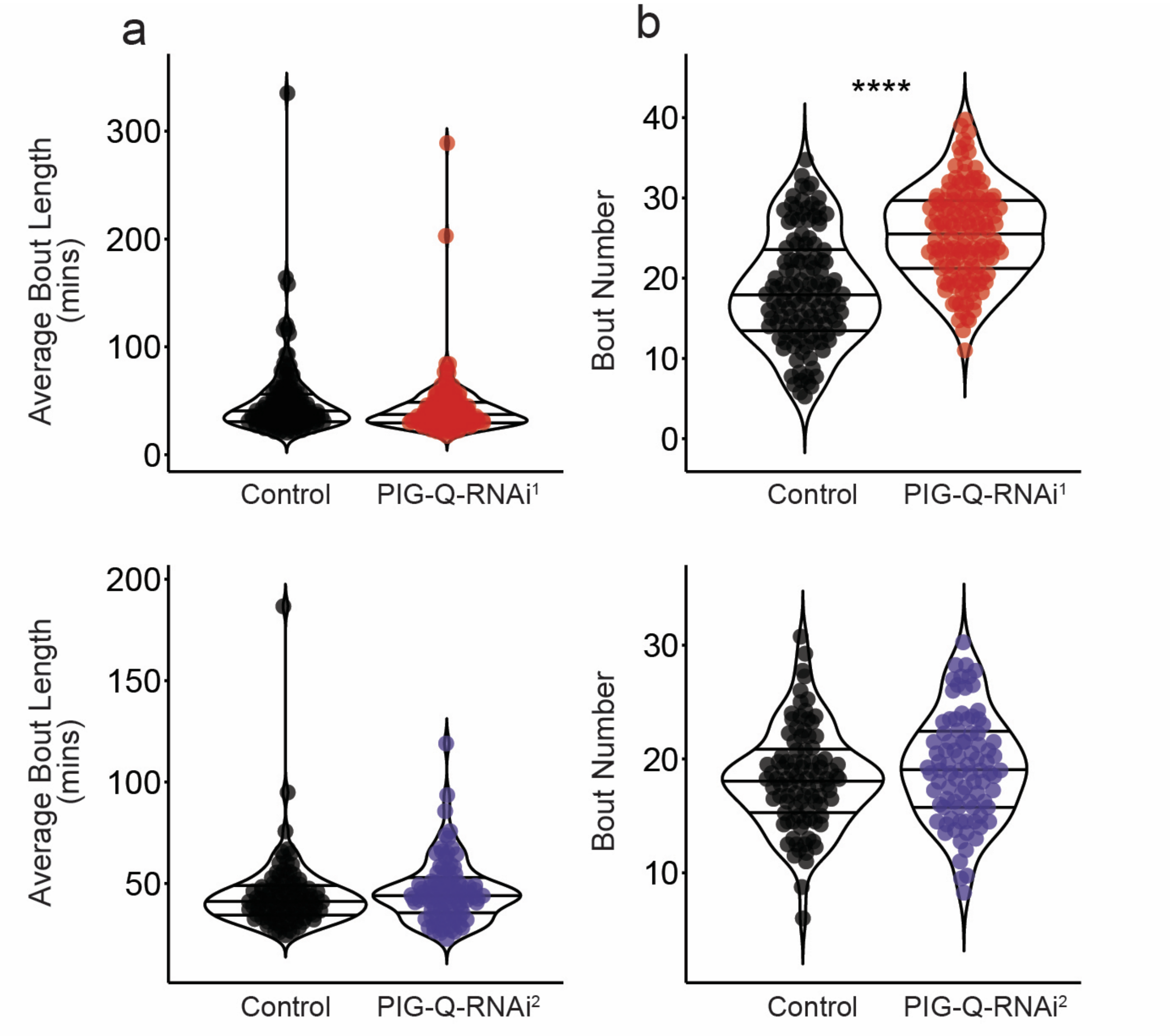

Figure S1. The effect of *PIG-Q* pan-neuronal knockdown on sleep traits. a, Knockdown of *PIG-Q* has no effect on bout length in two independent RNAi lines (t-test, *PIG-Q*-RNAi[1]: $t_{260} = 1.668$; P=0.0966; *PIG-Q*-RNAi[2]: $t_{184} = 0.8104$; P=0.4188). **b**, Knockdown of *PIG-Q* significantly increases bout number in one RNAi line, but has no effect in a second independent RNAi line (t-test, *PIG-Q*-RNAi[1]: $t_{260} = 8.949$; $P<0.0001$; *PIG-Q*-RNAi[2]: $t_{184} = 1.470$; P=0.1432). ****$P<0.0001$.

Figure S2

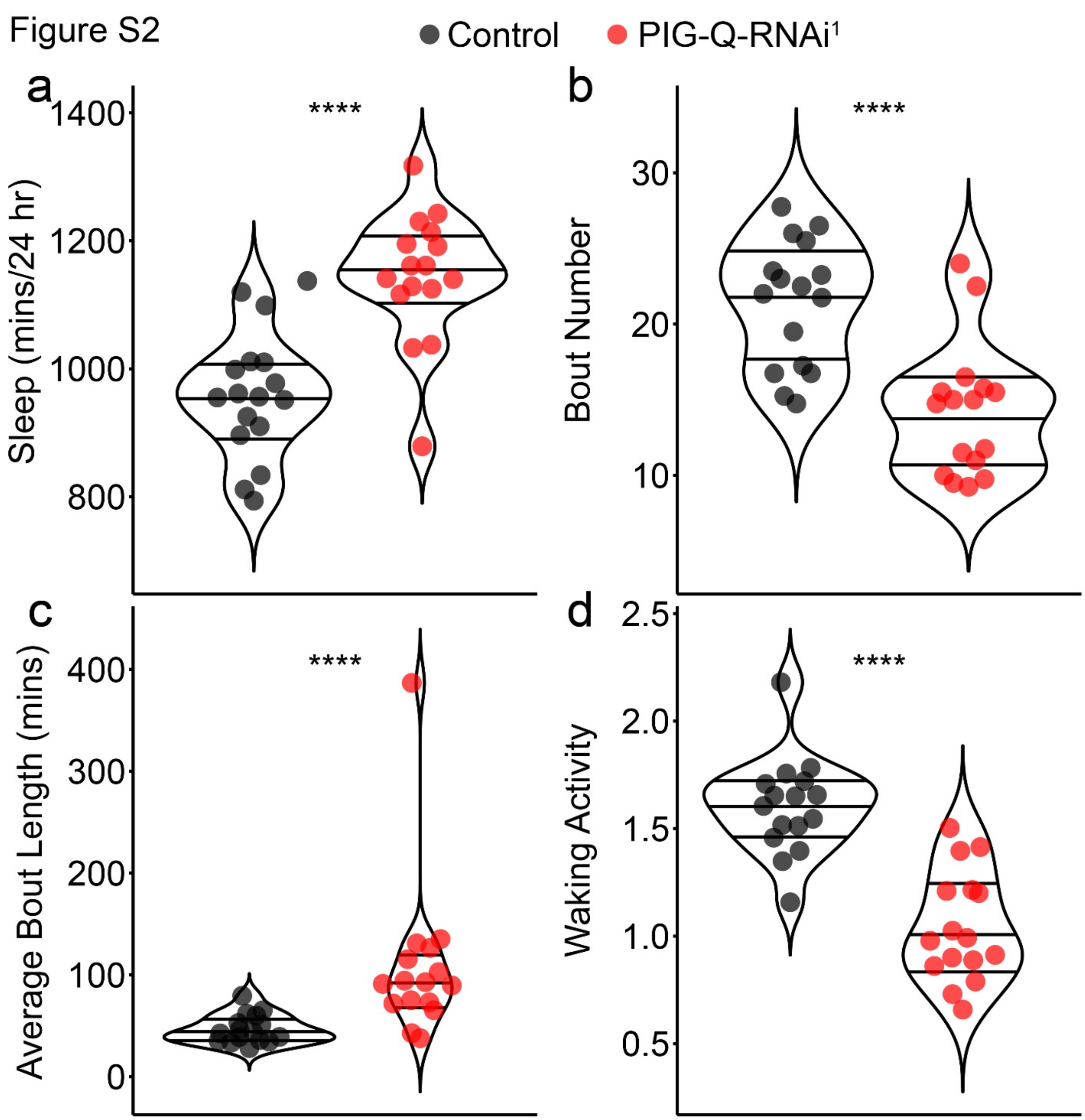

Figure S2. The effect of *PIG-Q* pan-neuronal knockdown on sleep traits in male flies. **a**, Knockdown of *PIG-Q* significantly increases sleep duration (t-test, $t_{30} = 5.708$; $P<0.0001$). **b**, Knockdown of *PIG-Q* significantly increases the length of each sleep episode (t-test, $t_{30} = 3.042$; $P<0.0001$), while significantly decreasing the number of sleep bouts (**c**, t-test, $t_{30} = 4.748$; $P<0.0001$). **d**, Knockdown of *PIG-Q* significantly decreases waking activity (t-test, $t_{30} = 6.596$; $P<0.0001$). ****$P<0.0001$.

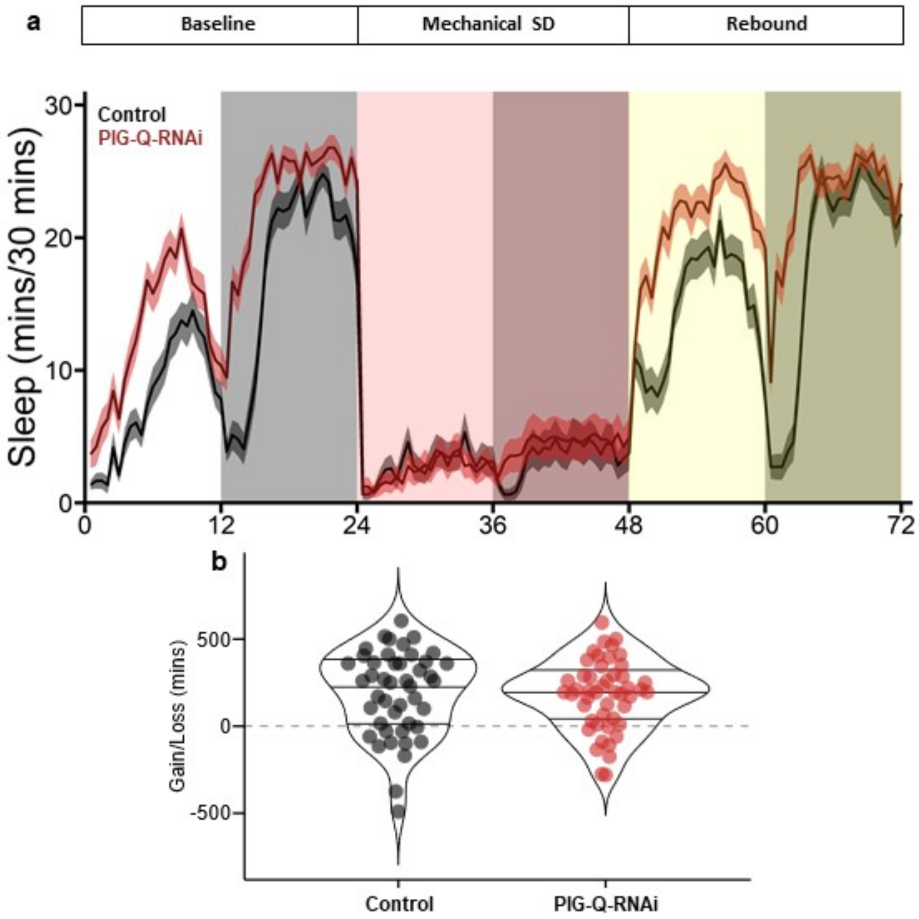

Figure S3. *PIG-Q* pan-neuronal knockdown has no effect on homeostatic sleep. a, Sleep profile of flies before (white), during (red), and after (yellow) 24 hrs of mechanical sleep deprivation. **b,** Knockdown of *PIG-Q* has no effect on sleep rebound. Minutes gained/lost are determined by measuring the acclimation period (white) and subtracting it from the rebound period (yellow) (t-test; $t_{55} = 7.387$; P=0.4816).

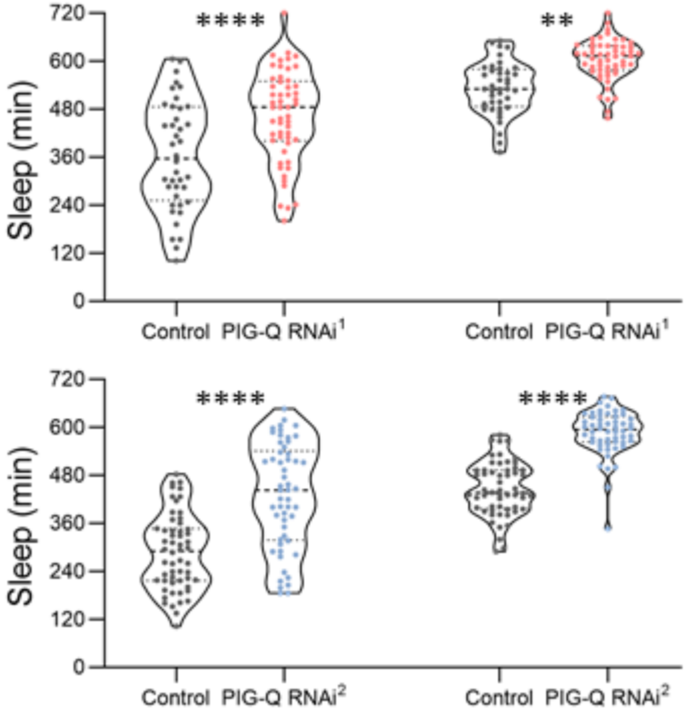

Figure S4. Sleep duration measurements of *PIG-Q* pan-neuronal knockdown in the DART system. **a**, Knockdown of *PIG-Q* significantly increases sleep in two independent RNAi lines (*PIG-Q*-RNAi[1]: $F_{1,184} = 34.75$, $P<0.0001$; *PIG-Q*-RNAi[2]: $F_{1,148} = 37.71$, $P<0.0001$), and occurs during both the day (*PIG-Q*-RNAi[1]: $P<0.0001$; *PIG-Q*-RNAi[2]: $P<0.0001$) and night (*PIG-Q*-RNAi[1]: $P=0.0014$; *PIG-Q*-RNAi[2]: $P<0.0001$). **$P<0.01$, ****$P<0.0001$.

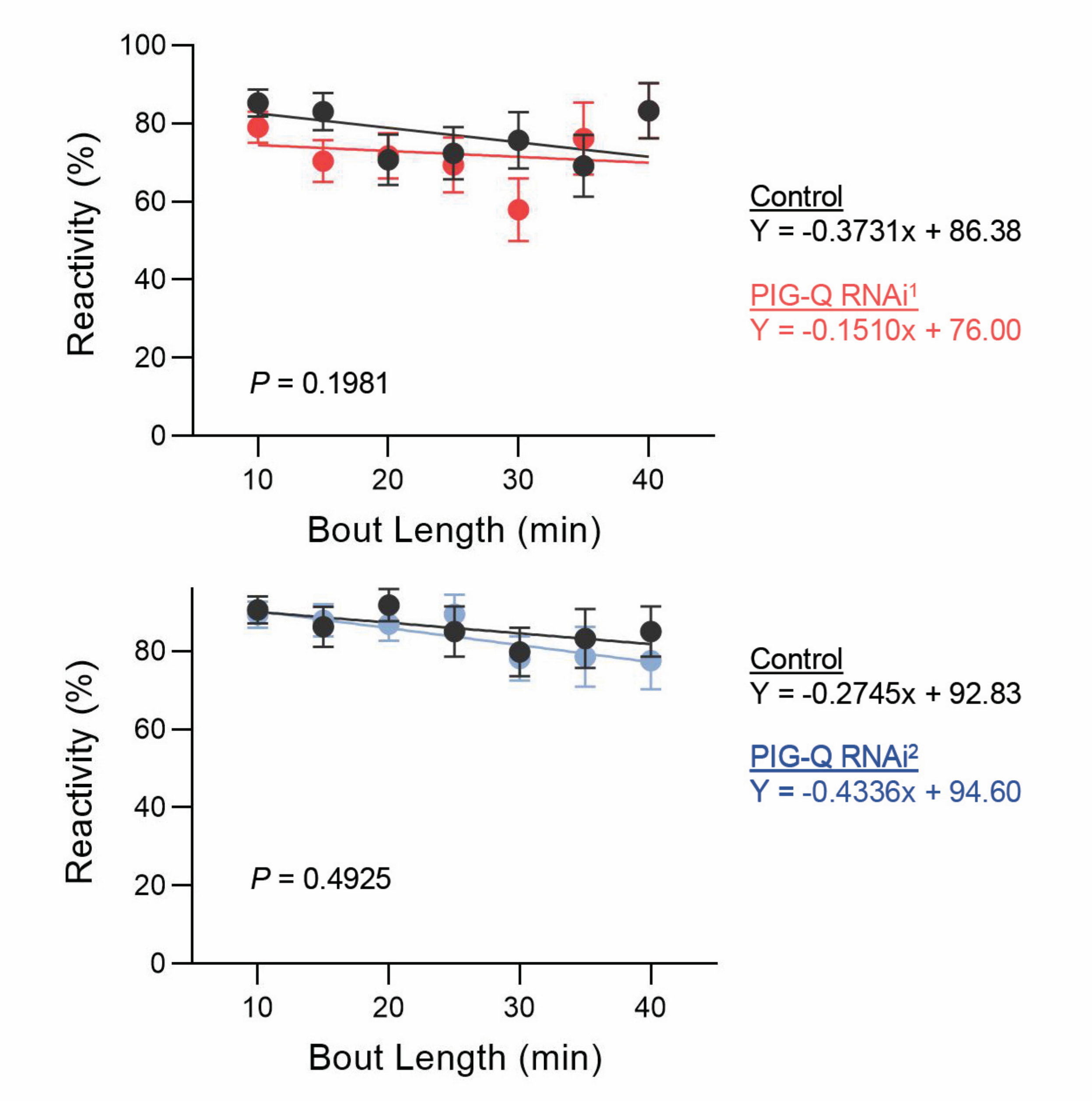

Figure S5. Daytime reactivity of pan-neuronal knockdown of *PIG-Q*. Knockdown of *PIG-Q* has no effect on daytime reactivity (ANCOVA with bout length as covariate, *PIG-Q*-RNAi[1]: $F_{1,715} = 0.4631$, P=0.4964; *PIG-Q*-RNAi[2]: $F_{1,637} = 0.3389$, P=0.5606).

Supplementary Tables

Table S1. Candidate target genes identified for the insomnia GWAS loci by our variant-to-gene mapping approach. GWAS loci are numbered according to [19]. Candidate regulatory proxy SNPs are reported with the r^2 value (in EUR population) to their respective sentinel SNPs. Candidate target genes are reported with their TPM (transcript per million) expression and percentile expression values from our bulk RNA-seq data in neural progenitor cells.

Table S2. RNAi screen of insomnia-associated orthologs. Raw sleep data from sleep screen includes stock numbers of each RNAi line used as well as the mean measurements of the other measurements of sleep including sleep duration, bout number, average bout length and waking activity.

Table S3. Localization of *PIG-Q*-RNAi long sleeping phenotype. Raw sleep data after knockdown of *PIG-Q* in multiple *Drosophila* neuronal subpopulations.

Table S4. Pan-neuronal knockdown of PIG pathway genes. Raw sleep data after pan-neuronal knockdown of PIG pathway genes.
